# Fulminant endophthalmitis after open globe injury by cat claw: two case reports and literature review

**DOI:** 10.1186/s12348-025-00487-5

**Published:** 2025-05-06

**Authors:** Ashley Y. Gao, Ameay V. Naravane, Michael A. Simmons, Tyler Looysen, Sandra Montezuma, Dara Koozekanani, Hossein Nazari

**Affiliations:** Department of Ophthalmology and Visual Neuroscience, University of Minnesota, 516 Delaware Street SE, 9-337, Minneapolis, MN 55455 USA

**Keywords:** Endophthalmitis, Animal-related ocular trauma, Open globe injury, Cat scratch

## Abstract

**Objective:**

To describe two patients with fulminant endophthalmitis after penetrating ocular injuries by cat claw and review the literature regarding animal-related endophthalmitis.

**Design:**

Case series.

**Participants:**

In the study period, 298 patients were identified with a diagnosis of endophthalmitis, of which two were identified in association with open globe injury by cat claw.

**Methods:**

All patients with endophthalmitis after cat claw open globe injury in an academic center in a 20-year period are reported. Clinical and laboratory presentations, medical and surgical treatment, and outcomes are described. A literature review is summarized.

**Exposure:**

Open globe injury by cat claw.

**Main outcome measures:**

Interventions and ocular anatomical and functional outcomes.

**Results:**

Case 1: A 27-year-old female sustained a penetrating injury of the left eye by a cat claw. The laceration was repaired the next day, and intravitreal antibiotics injections were given. She developed acute fulminant endophthalmitis the following day and underwent pars plana vitrectomy, anterior chamber washout, and intravitreal antibiotics injection. Cultures isolated *Propionibacterium acnes*. A retinal detachment was noted after 48 days, requiring a second pars plana vitrectomy and tamponade with sulfur hexafluoride gas. The retina remained attached. Visual acuity at 14 months follow-up was 20/200. Case 2: A 42-year-old male developed endophthalmitis two days after a penetrating injury of the right eye by a cat claw. Pars plana vitrectomy and intravitreal antibiotics injections were performed the same day. Cultures identified *Pasteurella multocida*. The patient progressed to panophthalmitis in 24 h and received intravenous antibiotics. He developed proliferative vitreoretinopathy with recurrent retinal detachments requiring multiple vitrectomies. His visual acuity was hand motions at 7 months follow-up.

**Conclusions and relevance:**

Open globe injuries caused by cat claw may result in hyperacute and acute endophthalmitis. *Propionibacterium acnes* and *Pasteurella multocida* were isolated from the two cases reported here. Despite immediate interventions, both patients developed retinal detachment and had poor final visual acuity. Our report reveals that endophthalmitis caused by animal trauma is rare with potentially devastating outcomes, thereby requiring timely diagnosis and treatment.

## Introduction

Ocular trauma is a significant cause of visual impairment, with an estimated world prevalence of approximately 19 million individuals having unilateral blindness or low vision as a result of eye injuries [1]. Animal-related injuries comprise a small subset of ocular trauma, and endophthalmitis following such injuries is infrequently reported. A review of English literature revealed twenty cases of post-traumatic endophthalmitis after a penetrating globe trauma associated with direct ocular penetration, or suspected ocular penetration, by an animal (Table 1), with only five known reported cases of direct cat claw-induced penetrating ocular trauma resulting in endophthalmitis [2–6]. 

**Table 1:** Animal-related causes of endophthalmitis

Animal	Pertinent History	Time Frame between Trauma and Presentation	Culture results	Surgeries performed following injury	First Reported Visual Acuity	Last Reported Visual Acuity	Retinal detachment following trauma?	Reference
Cat	Open globe injury by cat claw No evidence of cat scratch disease	Unknown	*Pseudomonas aeruginosa*	Sutured scleral wound Pars plana lensectomy and vitrectomy Scleral buckling and gas tamponade Second vitrectomy and retina reattachment	20/250	20/200	Yes	[2]
Cat	Open globe injury by cat claw No evidence of cat scratch disease	Same day	*Pasteurella multocida*	Lensectomy and vitrectomy	20/40	20/20	No	[3]
Cat	Open globe injury by cat claw No evidence of cat scratch disease	Same day	*Pasteurella multocida*	Sutured scleral wound and superficial lid laceration manual vitrectomy from the scleral wound	6/60	N/A (evisceration)	No	[4]
Cat	Open globe injury by cat claw No evidence of cat scratch disease	Unknown	*Pasteurella multocida*	Laceration repair Pars plana vitrectomy	Unknown	20/70 (uncorrected) 20/30 (corrected)	No	[5]
Cat	Open globe injury by cat claw No evidence of cat scratch disease	Same day	*Pasteurella multocida*,* CDC group EF-4*	Laceration repair Pars plana vitrectomy Repeat pars plana vitrectomy	Unknown	Light perception	No	[6]
Cat	Caused by cat bite No evidence of cat scratch disease	1 day	*Pasteurella multocida*	Lensectomy, anterior vitrectomy, diathermy around the perforated wound, and an encircling buckling procedure Second vitrectomy Third vitrectomy fluid-gas exchange and 360 scleral buckling with a silicone implant	Light perception with no light projection	40/200	Yes	[13]
Cat	Caused by cat bite No evidence of cat scratch disease	4 days	Alpha hemolytic *Streptococcus* and *Bacillus*	Exploration and repair of scleral puncture wound pars plana vitrectomy Cataract surgery	Hand motion	20/20	No	[14]
Cat	Caused by cat bite No evidence of cat scratch disease	Unknown	No organism was isolated from anterior chamber tap	inferior lid tear repair	Unknown	20/25	No	[15]
Cat	Caused by cat bite No evidence of cat scratch disease	unknown	Capnocytophaga Canimorsus	Laceration repair PPV Pars plana lensectomy and vitrectomy Scleral buckle and fluid-gas exchange	unknown	unknown	Yes	[16]
Cat	Mechanism of cat injury (ex. claw vs. bite) unknown Corneal laceration	2 days	Pasteurella multocida	Unknown	Light perception	20/40	Unknown	[12, 17]
Bee	Bee sting	2 days	Yeast	Pars plana vitrectomy, lensectomy, anterior chamber washout, corneal transplant and secondary lens implantation with penetrating keratoplasty, epiretinal membrane peel	No light perception	20/80	No	[18]
Bee	Bee sting	1 day	Pseudomonas aeruginosa and Aeromonas veronii	Exploration to perform full ocular examination and to exclude open globe injury Globe evisceration	20/200	N/A (evisceration)	Yes	[19]
Bee	Bee sting	3 days	*Aspergillus fumigatus*	Pars plana vitrectomy Pars plana lensectomy Revised vitrectomy with silicone oil tamponade	Hand motions close to face	Finger counting at 1 m	Yes	[20]
Bird		Unknown	Unknown	Case 1: primary repair, vitrectomy during primary repair, lensectomy Case 2: primary repair, vitrectomy during primary repair, lensectomy Case 3: primary repair	Case 1: 20/7,960 (HM) Case 2: 20/320 Case 3: 20/130	Case 1: 20/7,960 (HM) Case 2: 20/15,890 (LP) Case 3: 20/3,170 (CF 30 cm)	Unknown	[21]
Bird		1 week	Beta-hemolytic *Streptococcus*	Primary corneo-limbal tear repair Pars plana vitrectomy	6/24	6/12	No	[22]
Horse	Direct injury to the eye caused by a horse’s tail was suspected but not confirmed.	2 days	*Enterococcus casseliflavus*	Diagnostic vitrectomy Pars plana lensectomy and vitrectomy	Light perception	Light perception	Yes	[23]
Crab		2 days	*Streptococcus viridans*	Open globe repair, anterior chamber washout cataract extraction and intraocular lens implantation with posterior capsulectomy and pars plana vitrectomy	Hand motion (one month after trauma)	20/50	No	[24]
Cow	Injury occurred 3 days before symptom onset	unknown	*Listeria monocytogenes*	Vitreoretinal and cataract surgeries	Light perception	20/1000	Yes	[25]

We present two patients with direct cat claw-induced penetrating trauma to the eye that caused severe hyperacute and acute endophthalmitis with subsequent retinal detachments requiring repeated surgeries. Herein, we will discuss the clinical course, laboratory findings, and anatomic and visual outcomes of the patients and will review the literature for endophthalmitis caused by animal-induced penetrating ocular traumas.

## Methods

Epic SlicerDicer was used to select participants. Searching the organization database from 10/15/2003 to 10/15/2023, the following inclusion criteria were applied based on diagnosis: acute endophthalmitis, acute endophthalmitis of bilateral eyes, acute endophthalmitis of left eye, and acute endophthalmitis of right eye. Two hundred ninety eight patients were retrieved. An additional slice by diagnosis, “Contact with other mammals (ICD-10-CM: W55.* )”, was applied to retrieve 4 patients. A chart review was performed and found that two of these four patients were miscoded and, thus, were excluded from the study: one patient had a self-inflicted eye injury, and the other had suspected IV drug use-related endophthalmitis. Two patients were identified with a history of penetrating ocular injury from house cat claw and subsequent development of endophthalmitis. Clinical presentation, surgical treatment, antibiotic administration, culture results, and visual and structural outcomes over the course of several months were reported.

Additionally, a comprehensive literature search was performed in PubMed on 10/15/2023, with the following search keywords and strategies: endophthalmitis AND (((((((scratch) OR (bite)) OR (claw)) OR (sting)) OR (peck)) OR (tail)) OR (attack)) OR (beak). Out of ninety-six publications that were retrieved, 59 articles did not pertain to animal-associated causes of endophthalmitis and were excluded. Eight review articles were also excluded. Eight articles described endogenous endophthalmitis, mimicking endophthalmitis, subclinical endophthalmitis, or pseudo-endophthalmitis, and were excluded. Three articles that involved non-human subjects, one article describing a case of endophthalmitis with ambiguous etiology, one article describing a patient with cat scratch disease, and one non-English study were excluded. Three papers from one relevant review were included, in addition to the remaining 15 publications. A summary of these 18 publications is presented in Table 1.

## Case presentation

### Case 1

A 27-year-old female presented to the emergency department after being scratched in the left eye by a cat. Past ocular history was remarkable for high myopia and contact lens use. She had no relevant past medical history. On examination, visual acuity was 20/20 in the right eye and 20/400 in the left eye. Slit lamp examination of the right eye was unremarkable. The left eye was hypotonus and had an upper eyelid edema with a puncture wound just under the left eyebrow. She had a conjunctival and scleral laceration posterior to the nasal limbus and subconjunctival hemorrhage. The cornea was clear, but the anterior chamber was shallow with a hyphema, inflammatory fibrin strands, and total loss of the iris. Posterior segment examination revealed vitreous hemorrhage but an attached retina. Intravenous vancomycin and ceftazidime were administered. An exploratory peritomy revealed a 7 mm circumferential scleral laceration, posterior to the limbus, from ∼ 7 o’clock to 11 o’clock. Total aniridia was noted (there had been complete loss of the iris during the initial trauma), with remnants of uveal pigment at the borders of the laceration with no further uveal tissue prolapse. The laceration was repaired, and intravitreal ceftazidime and vancomycin were administered at the end of the procedure. Post-operatively, she received topical prednisolone and moxifloxacin.

**Fig. 1:**
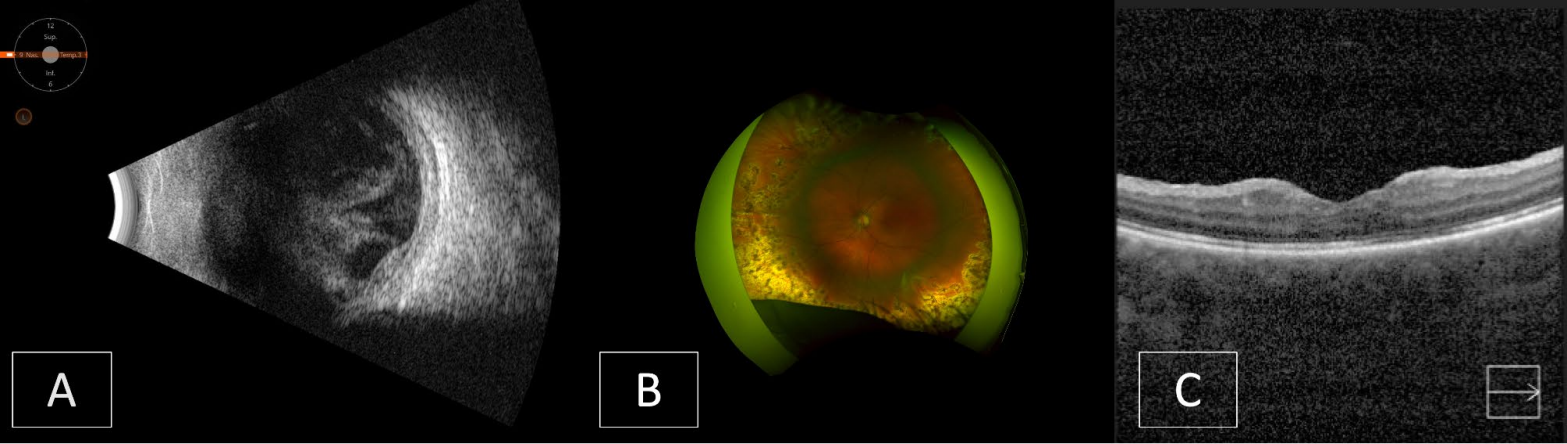
Case 1 images. **A**: B-scan ultrasound following the open globe repair. **B**: Optos image following the third pars plana vitrectomy. **C**: Optical coherence tomography one year post-trauma

On post-operative day 1, the vision was light perception with projection. Intraocular pressure was 9 mmHg. The cornea was mildly edematous with 2 + Descemet folds and a central endothelial plaque that spared the peripheral 1–2 mm of the cornea. An anterior chamber fibrinous reaction and hypopyon was noted. B-scan ultrasound showed an attached retina and increased vitreous opacities with web-shaped echodensities (Fig. 1A).

The patient was diagnosed with post-traumatic endophthalmitis and taken to the operating room on the same day for a pars plana vitrectomy, collection of vitreous samples for bacterial and fungal cultures, anterior chamber washout, and intravitreal injection of vancomycin, ceftazidime, and voriconazole. She received oral amoxicillin-clavulanic acid and topical prednisolone and moxifloxacin after the surgery. Vitreous culture isolated *Propionibacterium acnes*, but *s*usceptibility testing was not performed.

One day after the second surgery, visual acuity was light perception and intraocular pressure was 3 mmHg in the left eye. The slit lamp exam was notable for a 2 mm hypopyon. Posterior exam was limited due to vitreous haze. The retina was attached on B scan ultrasound. On postoperative day 8, the patient had a persistent anterior chamber inflammatory reaction and no view to the retina due to lens and vitreous opacities. Increasing vitreous opacities and an attached retina were confirmed with B scan ultrasound. A pars plana vitrectomy and lensectomy was performed, with peeling of inflammatory membranes and cryotherapy to the peripheral retina posterior to the sclerotomies.

Over the next few days, visual acuity remained at light perception in the left eye and the eye was hypotonic. Slit lamp examination showed an edematous cornea and resolving anterior chamber and vitreous inflammation. The retina was attached.

One month after the second pars plana vitrectomy surgery, visual acuity had improved to counting fingers at one foot, with an intraocular pressure of 13 mmHg. An inferior retinal detachment extending from 4 to 7 o’clock with attached macula and no apparent proliferative vitreoretinopathy (PVR) was noted. Optical coherence tomography (OCT) showed distorted fovea and outer retinal atrophy. She underwent a third pars plana vitrectomy, with the placement of a #4050 encircling buckle, endolaser, and 25% SF6 gas tamponade. The retina remained attached as gas resorbed in the postoperative period. Distance best corrected visual acuity improved to 20/400 one month after the last surgery (Fig. 1B).

Seven months after the initial trauma, the patient underwent a secondary intraocular lens placement by intrascleral haptic fixation (Yamane technique) and implantation of an artificial iris in the left eye. Additional procedures were performed later on, including a glaucoma tube shunt implantation and a Descemet’s stripping endothelial keratoplasty (DSAEK) for secondary glaucoma and corneal endothelial decompensation.

One year after the initial trauma, visual acuity was 20/200 in the left eye, and the retina was attached. OCT revealed mild epiretinal membrane and outer and diffuse atrophy, centrally and temporally (Fig. 1C).

### Case 2

A 42-year-old male presented to the Emergency Department with right eyelid swelling, pain, and vision loss two days after being scratched in the right eye by his cat. At presentation, the patient had a full-thickness puncture wound in the upper eyelid and a supposed full-thickness puncture wound at the superotemporal limbus. The pupil was irregular, but there was no tissue prolapse. The referring eye care provider deemed no primary repair was necessary. He had no relevant past medical or surgical history.

**Fig. 2:**
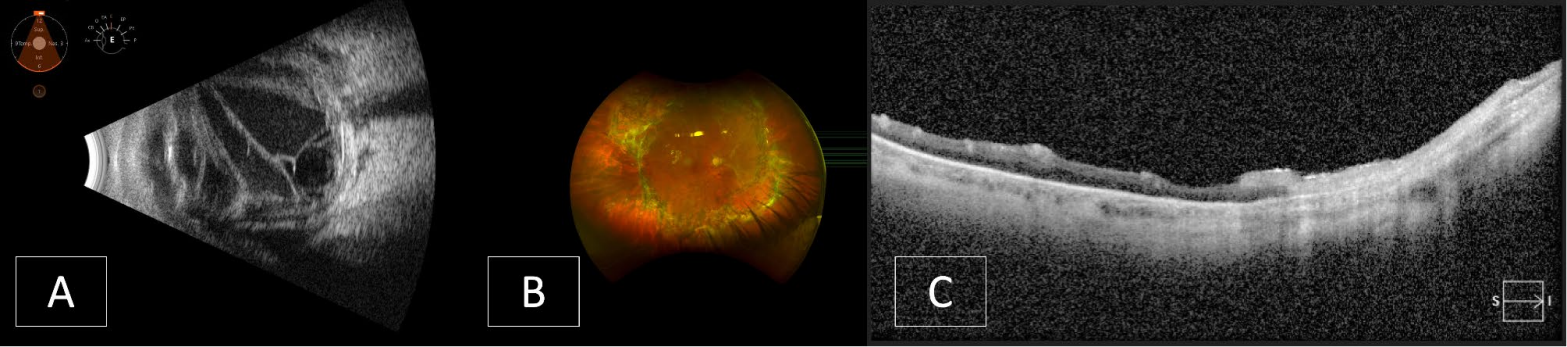
Case 2 images. **A**: B-scan ultrasound at presentation. **B**: Optos image following the third pars plana vitrectomy. **C**: Optical coherence tomography 6 months post-trauma

On exam, visual acuity was light perception with projection in the right eye and 20/50 in the left eye. The left eye was unremarkable and remained unchanged. Intraocular pressure was 29 mmHg in the right eye and 16 mmHg in the left eye. No afferent pupillary defect was noted by reverse. There was diffuse restriction of extraocular motility of the right eye. The right upper and lower eyelids were red and swollen. The conjunctiva was severely injected and chemotic with copious purulent discharges. The cornea had diffuse haziness, and anterior chamber had 3 + cells and a hypopyon. The iris was minimally reactive with irregular diffuse posterior synechiae and a fibrin membrane over the anterior lens capsule. There was no view to the fundus due to dense vitritis. B-scan ultrasound showed fibrinous membranes and debris in the vitreous cavity with a possible shallow serous retinal detachment (Fig. 2A). There was a positive T-sign suggestive of posterior scleritis. The patient was diagnosed with acute post-traumatic endophthalmitis and underwent a 23-gauge pars plana vitrectomy with vitreous sampling for microbiology cultures and intravitreal injection of ceftazidime, vancomycin, and amphotericin B. Intravenous antibiotics were administered during the surgery. Vitreous culture identified *Pasteurella multocida*, sensitive to penicillin and ceftriaxone. Post-operatively, the patient was started on topical prednisolone and moxifloxacin.

On post-operative day one, visual acuity was light perception in the right eye and intraocular pressure was 12 mmHg. The right eye had corneal edema, severe anterior chamber inflammation, and vitreous haze limiting view to the retina. In addition, the presence of proptosis, abnormal extraocular movement, and CT scan findings raised concerns for right orbital cellulitis and panophthalmitis. The patient was started on intravenous (IV) vancomycin, cefepime, and metronidazole that were then switched to IV ceftriaxone and IV moxifloxacin based on the recommendation from infectious disease service.

Over the next two weeks, corneal and anterior chamber inflammation improved, and the extraocular movement normalized. Intraocular pressure remained low. There was still no view to the retina due to vitreous opacities. A B-scan ultrasound examination revealed vitreous bands consistent with contractile inflammatory membranes and a retinal detachment. A pars plana vitrectomy, lensectomy, and 5,000 centistokes (cs) silicone oil tamponade surgery was performed. The retina remained attached, and vision improved during the postoperative course; however, a recurrent rhegmatogenous retinal detachment with PVR formation was noted at two months follow up. The patient underwent a repeat pars plana vitrectomy, membrane peel, and retinectomy, with silicone oil tamponade (Fig. 2B).

Six months after the initial trauma, visual acuity was counting fingers at two feet, with loss of peripheral vision. The patient remained aphakic. OCT revealed no subretinal fluid, temporal atrophy, an epiretinal membrane, and possible resolution of a small subretinal perfluoro-N-octane bubble inferiorly (Fig. 2C). Plans for scleral fixation of an intraocular lens were discussed; however, given poor visual prognosis, the decision was made to observe.

## Discussion

Here we report two cases of open globe injury secondary to cat scratch: a 27-year-old female who had a penetrating corneoscleral laceration from a house cat claw resulting in hyperacute *Propionibacterium acnes* endophthalmitis, and a 42-year-old male who similarly had a penetrating ocular laceration from cat claw and developed acute *Pasteurella multocida-*related panophthalmitis. Despite timely diagnosis and management, both patients had a protracted course that was complicated with recurrent retinal detachments requiring repeated pars plana vitrectomy surgeries. In both cases, final visual acuity was limited due to macular atrophy, likely due to recurrent macula-off retinal detachment, the lengthy course of intraocular inflammation, and possibly toxicity from bacterial by-products.

The vitreous sample culture of the first patient grew *P. acnes*, which is typically considered a low-virulence bacteria, often associated with slow and indolent post-cataract surgery endophthalmitis [7]. Our patient, in contrast, developed a fulminant endophthalmitis within the first 48 h of injury. *P. acnes* is an unusual cause of hyperacute and acute severe post-traumatic endophthalmitis [8], and fulminant endophthalmitis caused by *P. acnes* has not been reported in the literature. Moreover, *P. acnes* can be challenging to culture due to its slow growth rate [8]. It is possible that the fulminant course occurred in our patient because the eye was inoculated with a high bacterial load. It is also possible that the endophthalmitis was polymicrobial and a second organism was never identified. The second patient grew *P. multocida*, a gram-negative zoonotic coccobacillus that is isolated from over 90% of healthy cats and is implicated in 75% of all infected cat bites [9, 10]. 

In both cases, exogenous endophthalmitis occurred as a result of direct ocular penetration by a cat claw, distinct from the cat scratch disease that is classically a neuroretinitis from Bartonella henselae bacteria, usually inoculated through a skin scratch by a cat bite or claw in a location away from the eye. Post-traumatic endophthalmitis accounts for 2–15% of all endophthalmitis cases, and animal-related injuries resulting in endophthalmitis represent a small subset of post-traumatic endophthalmitis [11]. The visual outcomes of endophthalmitis associated with trauma can be devastating, as demonstrated in a retrospective study, which found that five out of six eyes of trauma-associated endophthalmitis lost useful vision [12]. 

A review of English literature with the criteria detailed in the methods section revealed twenty cases of post-traumatic endophthalmitis after a penetrating globe trauma associated with direct ocular penetration, or suspected ocular penetration, by an animal (Table 1). Of these 20 cases, 50% involved cat-related injuries, but other animals such as dogs, bird, crab, horse, caw, and bees have also been reported to cause penetrating eye injury and subsequent endophthalmitis. The time frame between animal trauma and presentation with symptoms and signs of endophthalmitis ranged from the same day to one week after the trauma. Fourteen out of 20 reported patients needed vitrectomy surgery and nine of these patients required a lensectomy. Microbiology cultures from ocular samples revealed *Pasteurella* species in six, *Streptococcus* species in three (one of which also identified *Bacillus*), and *Pseudomonas* species in two (one of which also identified *Aeromonas*) patients (Table 1). Visual outcomes varied widely from 20/20 to light perception, depending on the mechanism and severity of injury. Three patients required either an evisceration or enucleation. Seven out of 16 patients with animal-related globe penetration and endophthalmitis developed retinal detachment, and three of these seven cases were associated with cat trauma. The fact that both of our patients had endophthalmitis and retinal detachment demonstrates how cat-related open globe injuries and endophthalmitis may carry a poor prognosis.

The rate of endophthalmitis after penetrating eye trauma is 1–18%, and untreated endophthalmitis often results in irreversible blindness within hours or days [11]. Therefore, it is imperative for clinicians to closely monitor patients with open globe injuries caused by animal bite or claw for the development of endophthalmitis. Ocular cultures and prophylactic use of broad-spectrum intraocular antibiotics (such as intravitreal vancomycin or ceftazidime or intracameral moxifloxacin) at the time of the primary repair should be strongly considered. Prophylactic administration of antifungals may or may not be clinically defensible: the rate of exogenous animal/insect-related fungal endophthalmitis is likely low, as only two publications in English literature have reported post-traumatic fungal endophthalmitis, and these occurred after bee stings.

In summary, we reported two cases of hyperacute and severe endophthalmitis after open globe traumas by cat claw and reviewed medical literature for animal trauma-associated endophthalmitis. Our two patients endured a complicated course associated with recurrent retinal detachments. Despite successful anatomic outcomes, both had limited visual recovery. Animal-related open globe injuries with subsequent endophthalmitis are rarely reported in the literature but are commonly associated with a complicated course and a guarded visual prognosis. Our experience suggests a possible benefit for prophylactic intraocular antibiotics at the time of primary repair after animal-related open globe trauma. Moreover, it would be beneficial to consider culturing the source of the infection, in our case the cat claw, particularly due to the concern for polymicrobial infection.

## Data Availability

No datasets were generated or analysed during the current study.
